# Effective anti-mycobacterial treatment for BCG disease in patients with Mendelian Susceptibility to Mycobacterial Disease (MSMD): a case series

**DOI:** 10.1186/s12941-022-00500-y

**Published:** 2022-03-01

**Authors:** Seyed Alireza Mahdaviani, Mazdak Fallahi, Mahnaz Jamee, Majid Marjani, Payam Tabarsi, Afshin Moniri, Parisa Farnia, Zahra Daneshmandi, Nima Parvaneh, Jean-Laurent Casanova, Jacinta Bustamante, Davood Mansouri, Ali Akbar Velayati

**Affiliations:** 1 Pediatric Respiratory Diseases Research Center, National Research Institute of Tuberculosis and Lung Diseases (NRITLD), Shahid Beheshti University of Medical Sciences, Tehran, Iran; 2 Pediatric Nephrology Research Center, Research Institute for Children’s Health, Shahid Beheshti University of Medical Sciences, Tehran, Iran; 3 Pediatric Infections Research Center, Research Institute for Children’s Health, Shahid Beheshti University of Medical Sciences, Tehran, Iran; 4 Clinical Tuberculosis and Epidemiology Research Centre, National Research Institute of Tuberculosis and Lung Diseases (NRITLD), Shahid Beheshti University of Medical Sciences, Tehran, Iran; 5 Mycobacteriology Research Centre (MRC), National Research Institute of Tuberculosis and Lung Diseases (NRITLD), Shahid Beheshti University of Medical Sciences, Tehran, Iran; 6 Division of Allergy and Clinical Immunology, Department of Pediatrics, Tehran University of Medical Sciences, Tehran, Iran; 7 Laboratory of Human Genetics of Infectious Diseases, Necker Branch, UMR 1163, Necker Hospital for Sick Children, INSERM, University of Paris, Imagine Institute, 75015 Paris, EU France; 8 St. Giles Laboratory of Human Genetics of Infectious Diseases, Rockefeller Branch, The Rockefeller University, New York, NY USA; 9 Howard Hughes Medical Institute, New York, NY USA; 10 Center for the Study of Primary Immunodeficiencies, Necker Hospital for Sick Children, AP-HP Paris, EU France

**Keywords:** MSMD, BCG-osis, IFN-γ, Anti-mycobacterial agents, Inborn error of immunity

## Abstract

**Background:**

Post-vaccination BCG disease typically attests to underlying inborn errors of immunity (IEIs), with the highest rates of complications in patients with Mendelian susceptibility to mycobacterial disease (MSMD). However, therapeutic protocols for the management of BCG-osis (disseminated) and persistent BCG-itis (localized) are still controversial.

**Methods:**

Twenty-four Iranian patients with MSMD (BCG-osis or BCG-itis), followed from 2009 to 2020 in Tehran, were included in the study. Their medical records were retrospectively reviewed for demographics, clinical features, laboratory findings, and molecular diagnosis. The therapeutic protocol sheets were prepared to contain the types and duration of anti-mycobacterial agents.

**Results:**

BCG disease either as BCG-itis (33.3%) or BCG-osis (66.7%) was confirmed in all patients by positive gastric washing test (54.2%), microbial smear and culture (58.3%), or purified protein derivative (PPD) test (4.2%). The duration between BCG-osis onset and MSMD diagnosis was 21.6 months.

All except three patients were initiated on second-line anti-mycobacterial agents with either a fluoroquinolone (levofloxacin: 15 mg/kg/day, ciprofloxacin: 20 mg/kg/day, ofloxacin: 15 mg/kg/day), aminoglycoside (amikacin: 10–15 mg/kg/day, streptomycin: 15 mg/kg/day), and/or macrolide (clarithromycin: 15 mg/kg/day) along with oral rifampin (10 mg/kg/day), isoniazid (15 mg/kg/day), and ethambutol (20 mg/kg/day). Three patients showed a clinical response to rifampin, despite in vitro resistance. Fourteen (58.3%) patients received also adjuvant subcutaneous IFN-γ therapy, 50 µ/m^2^ every other day. At the end of survey, most patients (n = 22, 91.7%) were alive and two patients died following BCG-osis and respiratory failure.

**Conclusions:**

We recommend the early instigation of second-line anti-mycobacterial agents in MSMD patients with BCG disease.

**Supplementary Information:**

The online version contains supplementary material available at 10.1186/s12941-022-00500-y.

## Introduction

Bacillus-Calmette Guérin (BCG) vaccines are live attenuated substrains of *Mycobacterium bovis* that are used globally for the prevention of tuberculosis (TB). Since 1974, as part of the national vaccination policy, all Iranian children receive a single dose of BCG at birth or first post-natal visit [1]. The most common reported adverse effects of the BCG vaccine are lymphadenitis and local–regional (BCG-itis) or disseminated BCG infection (BCG-osis) [2]. BCG-vaccine-derived adversities may point out an underlying inborn error of immunity (IEI), with the highest rates of complications and mortality rate among Mendelian susceptibility to mycobacterial disease (MSMD) and severe combined immunodeficiency (SCID) patients, respectively [3–6]. In such patients, an early anti-mycobacterial regimen is life-saving [7]. However, the therapeutic protocols for the management of BCG vaccine complications are still controversial. All BCG strains are resistant to pyrazinamide while sensitivity to other anti-mycobacterial agents is variable [8]. Adjuvant therapy with recombinant interferon-gamma (rIFN-γ) has shown to be effective in BCG-osis associated with some types of MSMD and chronic granulomatous disease (CGD) [9, 10]. In patients diagnosed with autosomal recessive (AR) complete IFN-γ receptor 1 or 2 deficiency (or AR STAT1 deficiency) or SCID with available matched donors, allogenic hematopoietic stem cell transplantation (HSCT) can be a curative option [4, 11]. However, in MSMD patients, due to the recurrent infections and high serum levels of IFN-γ, the HSCT may lead to unfavorable outcomes [12]. Recently, the transduction of lentiviral vectors expressing the human IFN-γR1 cDNA has been able to restore the IFN-γ pathway without adverse effects of HSCT [13, 14]. Nevertheless, even in patients who are candidates for HSCT or gene therapy, the infections should be under control which relies on prior treatment with an anti-mycobacterial regimen [15]. Finally, the molecular genetic etiology is not known when BCG disease is diagnosed and therapeutic decisions must be made promptly. The proposed anti-mycobacterial regimen for BCG disease is most commonly composed of a combination of isoniazid, rifampin, and ethambutol with associated fluoroquinolones, macrolides, or aminoglycosides, prescribed mainly based on the clinical response or in vitro susceptibility profile [16, 17].

In the present study, we aim to investigate an appropriate therapeutic regimen for the BCG vaccine complications in patients with MSMD.

## Methods

The study was performed in an 11-year-period in the National Research Institute of Tuberculosis and Lung Diseases (NRITLD), Masih Daneshvari Hospital, Tehran, Iran, which is the national referral center for tuberculosis and BCG vaccine complications. All patients with both BCG-osis and clinical or molecular diagnosis of MSMD were included in the study. The diagnosis of BCG-osis/BCG-itis and underlying MSMD was made according to the criteria of the European Society for Immunodeficiencies (ESID) working party. The immunologic profiles of patients were determined by the measurement of serum immunoglobulin levels (IgG, IgA, IgM, and IgE), lymphocyte subsets, human immunodeficiency virus (HIV) serology, nitro-blue tetrazolium (NBT) test, lymphocyte transformation test (LTT), inflammatory markers such as erythrocyte sedimentation rate (ESR) and C-reactive protein (CRP). The purified protein derivative (PPD) test, gastric washing, and smear and cultures for mycobacteria species were performed as confirmatory tests. The DNA samples were sent to the Laboratory of Human Genetics of Infectious Diseases and for twenty patients, genetic studies by whole-exome sequencing (WES) were performed to identify underlying genetic defects. For the other four patients (P17, P18, P19, P23) DNA samples were not available. The therapeutic protocol sheets were prepared to contain the characteristics of anti-mycobacterial agents prescribed for each patient based on the proposed dosage and duration in reference books [16, 17]. A questionnaire was prepared and filled with patients’ data including demographics, clinical features, and laboratory findings. The drug susceptibility test was performed using the QIAamp® DNA kits (QIAGEN company, Germany). The isolates were tested for susceptibility to rifampin, Isoniazid, ethambutol, amikacin, kanamycin, and ciprofloxacin using molecular method. The proportion of pyrazinamide assay was used to differentiate microbial resistance between the BCG and TB species. All statistical analyses were performed using SPSS software (v. 26.0, Chicago, IL).

## Results

### Demographics

A total number of twenty-four MSMD patients with BCG-osis or BCG-itis were enrolled in the study from 2009 to 2020. Fifteen patients (P1-P15) were reported in our previous study [18]. The male–female ratio was 1:1 and the median (IQR) age at the time of the study was 9.8 (6.7–12.0) years. Most patients were Persian (n = 8, 33.3%), Baluch (n = 7, 29.2%), and Azeri (n = 5, 20.8%), mainly from Sistan and Baluchestan (29.2%) and Azerbaijan (20.8%) provinces. Most patients (n = 20, 83.3%) had consanguineous parents. All patients except two siblings (P20 and P21) were sporadic. BCG vaccination was inoculated in 23 out of 24 patients mainly at birth in 15 (62.5%) patients or the median (IQR) age of 0.6 (0.1–3.5) months in other patients. The median (IQR) age at the onset of BCG disease was 2.4 (0.4–3.6) months. The clinical diagnosis of MSMD was achieved at the median (IQR) age of 2.0 (0.4–5.8) years. For nineteen patients, the genetic study confirmed responsible genetic mutations at *IL12RB1* (n = 10, 41.7%), *IL12B* (n = 6, 25.0%), *IL23R* (n = 2, 8.3%), and *TYK2* (n = 1, 4.2%) genes. P24 had a heterozygous variant in *IL12RB1*, but she died before assessment of IL12RB1 protein expression. Table 1 shows the detailed demographic and clinical characteristics of patients.

**Table 1:** Summary of demographic and clinical features of MSMD patients

Patient No	Mutated Gene	Sex	Ethnicity	Age at BCG disease (month)	Age at MSMD diagnosis (year)	BCG disease type	Coinfections	Other comorbidities	References
1	*IL23R*	Male	Azeri	0.48	5.00	Disseminated	No	Lung involvement, Splenomegaly, Enteropathy	[18, 33]
2	*IL12B*	Female	Baluch	0.36	2.05	Disseminated	*Candida*	No	[18]
3	*IL12RB1*	Female	Persian	0.72	2.01	Disseminated	*Salmonella, Klebsiella*	Lung involvement	[18, 24]
4	*IL12B*	Female	Baluch	0.48	0.04	Disseminated	*Salmonella, Candida*	No	[18]
5	*IL12RB1*	Male	Persian	1.32	0.11	Disseminated	No	No	[18]
6	*IL12B*	Female	Baluch	6.00	3.00	Disseminated	*Candida*	No	[18]
7	*IL23R*	Male	Persian	3.60	7.00	Disseminated	No	No	[18]
8	*IL12B*	Female	Baluch	0.00	12.00	Disseminated	No	Vasculitis	[18]
9	*IL12RB1*	Female	Kurd	0.36	0.05	Disseminated	*Salmonella*	Splenomegaly	[18]
10	*TYK2*	Male	Baluch	0.36	7.00	Disseminated	No	Unilateral paresthesia, Lung involvement	[18, 34]
11	*IL12RB1*	Male	Persian	25.20	6.00	Disseminated	*Salmonella*	No	[18]
12	*IL12RB1*	Male	Persian	0.36	0.08	Disseminated	No	Splenomegaly	[18]
13	*IL12RB1*	Female	Persian	0.48	0.04	Disseminated	No	Splenomegaly	[18]
14	*IL12B*	Male	Baluch	0.36	7.00	Disseminated	No	Splenomegaly	[18]
15	*IL12RB1*	Male	Persian	4.80	2.00	Localized	No	No	(18)
16	*IL12B*	Male	Baluch	3.60	9.20	Localized	No	No	Unpublished
17	N/D	Male	Afghan	7.20	1.20	Localized	*Klebsiella, Enterobacter*	No	Unpublished
18	N/D	Male	Azeri	2.40	1.00	Localized	No	Splenomegaly, Enteropathy	Unpublished
19	N/D	Female	Afghan	2.40	2.50	Localized	*Candida*	Enteropathy, Atopy	Unpublished
20	*IL12RB1*	Female	Azeri	3.00	0.30	Localized	No	Septic arthritis, Atopy, Lung involvement, Splenomegaly	Unpublished
21	*IL12RB1*	Female	Azeri	42.00	3.00	Disseminated	No	Enteropathy, Atopy, Lung involvement	Unpublished
22	*IL12RB1*	Female	Azeri	3.60	2.80	Localized	No	No	Unpublished
23	N/D	Male	Persian	3.60	0.80	Localized	No	Splenomegaly	Unpublished
24	N/D	Female	Afghan	3.60	1.25	Disseminated	No	Splenomegaly, Lung involvement	Unpublished

### Clinical and laboratory features

The case presentations and summary of laboratory findings are described in Additional files 1 and 2. BCG complications either BCG-itis (n = 8, 33.3%) or BCG-osis (n = 16, 66.7%) forms with *Mycobacterium bovis-*BCG subspecies were confirmed in all patients by positive gastric washing test (n = 13, 54.2%), microbial smear and culture (n = 14, 58.3%), or PPD (n = 1, 4.2%). Other organ involvements included splenomegaly (n = 9, 37.5%), respiratory infections (n = 6, 25.0%), enteropathy (n = 4, 16.7%), atopy (n = 3, 12.5%), septic arthritis (n = 1, 4.2%), and leukocytoclastic vasculitis (n = 1, 4.2%). Besides BCG-osis, concomitant infections were reported with *Candida* species in four patients (16.7%), *Salmonella* in four patients (16.7%), *Klebsiella* in two patients (8.3%), and *M. tuberculosis* and *Enterobacter* each in one patient (4.2%).

Susceptibility profile for the *M. bovis-*BCG to various first- and second-line anti-mycobacterial agents including rifampicin, isoniazid, ethambutol, amikacin/kanamycin, and ciprofloxacin was evaluated in 14 patients (Table 2). The sensitivity to anti-TB drugs was as follows: Isoniazid (12 of 14, 85.7%), Ethambutol (11 of 14, 78.6%), Rifampin (8 of 14, 57.1%), Amikacin/Kanamycin (8 of 14, 57.1%), and Ciprofloxacin (7 of 14, 50.0%).

**Table 2:** Antibiotic susceptibility, therapeutic modalities, and outcomes

Pt No	Susceptibility profile					First-line therapy	Final therapy	IFN-γ	Surgery	Outcome (Cause of death)
	RIF	INH	ETB	AMIK	CIP					
1	R	S	N/D	N/D	N/D	Isoniazid, Rifampin, Ethambutol, Ofloxacin, Clarithromycin	Isoniazid, Rifampin, Ethambutol, Ofloxacin, Dapsone, Cycloserine	Yes	Abdominal surgery, Lymphadenectomy	Deceased (Respiratory failure)
2	S	S	S	S	S	Isoniazid, Rifampin, Ethambutol, Amikacin, Clarithromycin (~ Pyrazinamide)	Isoniazid, Rifampin, Ethambutol, Amikacin, Ofloxacin	Yes	No	Alive
3	R	R	S	N/D	N/D	Isoniazid, Rifampin, Ethambutol, Clarithromycin, Ofloxacin (~ Amikacin and Ethionamide)	Levofloxacin, Clarithromycin, Ethambutol, Cycloserine, Ethionamide, Linezolid, Clofazimine (~ Amikacin and Prothionamide)	Yes	Abdominal surgery	Alive
4	N/D	N/D	N/D	N/D	N/D	Isoniazid, Rifampin, Ethambutol, Clarithromycin, Ciprofloxacin	Isoniazid, Rifampin, Ethambutol, Clarithromycin, Levofloxacin, Amikacin	Yes	Lymphadenectomy	Alive
5	N/D	N/D	N/D	N/D	N/D	Isoniazid, Rifampin, Ethambutol, Clarithromycin, Ofloxacin	Isoniazid, Rifampin, Ethambutol, Clarithromycin, Ofloxacin	No	No	Alive
6	S	S	S	N/D	N/D	Rifampin, Ethambutol, Pyrazinamide, Clarithromycin	Isoniazid, Rifampin, Ethambutol, Levofloxacin	Yes	No	Alive
7	S	S	S	S	S	Ethambutol, Levofloxacin, Prothionamide, Cycloserine (~ Amikacin)	Isoniazid, Ethambutol, Levofloxacin	No	No	Alive
8	N/D	N/D	N/D	N/D	N/D	Isoniazid, Rifampin, Ethambutol, Amikacin, Clarithromycin, Ofloxacin (~ Dapsone, Clofazimine)	Ethambutol, Amikacin, Clarithromycin, Levofloxacin, Fluvoxamine, Prothionamide, Cycloserine	Yes	Abdominal surgery, lymphadenectomy	Alive
9	S	S	N/D	N/D	N/D	Isoniazid, Rifampin, Ethambutol, Clarithromycin, Ciprofloxacin	Isoniazid, Rifampin, Ethambutol, Clarithromycin, Ciprofloxacin	Yes	Lymphadenectomy	Alive
10	R	R	S	S	R	Isoniazid, Rifampin, Ethambutol, Amikacin, Ofloxacin, Clarithromycin (~ Streptomycin, Prothionamide)	Amikacin, Levofloxacin, Cycloserine, Ethionamide, Clofazimine, Meropenem, Co-Amoxiclav	Yes	Abdominal surgery (Psoas abscess drainage)	Alive
11	R	S	S	S	S	Isoniazid, Rifampin, Ethambutol, Pyrazinamide	Isoniazid, Rifampin, Ethambutol, Levofloxacin, Co-Trimoxazole (~ Amikacin)	Yes	No	Alive
12	N/D	N/D	N/D	N/D	N/D	Isoniazid, Rifampin, Ethambutol, Clarithromycin (~ Cycloserine)	Isoniazid, Rifampin, Ethambutol, Clarithromycin	Yes	No	Alive
13	N/D	N/D	N/D	N/D	N/D	Isoniazid, Rifampin, Ethambutol, Ofloxacin, Clarithromycin	Isoniazid, Rifampin	No	Lymphadenectomy	Alive
14	N/D	N/D	N/D	N/D	N/D	Isoniazid, Rifampin, Ethambutol, Clarithromycin, Ofloxacin	Isoniazid, Rifampin, Ethambutol, Clarithromycin, Ofloxacin	No	No	Alive
15	N/D	N/D	N/D	N/D	N/D	Isoniazid, Rifampin, Ethambutol, Clarithromycin	Isoniazid, Rifampin, Ethambutol, Clarithromycin	Yes	No	Alive
16	N/D	N/D	N/D	N/D	N/D	Isoniazid, Rifampin, Ethambutol, Clarithromycin	Isoniazid, Rifampin, Ethambutol, Amikacin, Levofloxacin	No	Lymphadenectomy	Alive
17	S	S	S	S	S	Isoniazid, Rifampin, Ethambutol, Levofloxacin, Amikacin (~ Meropenem, Linezolid)	Isoniazid, Rifampin, Ethambutol, Levofloxacin, Amikacin, Cycloserine	Yes	Central vein catheterization	Alive
18	R	S	S	S	S	Isoniazid, Rifampin, Ethambutol	Rifampin, Levofloxacin, Cycloserine, Isoniazid, Ethambutol, Linezolid	No	Lymphadenectomy	Alive
19	N/D	N/D	N/D	N/D	N/D	Isoniazid, Rifampin, Ethambutol, Clarithromycin, Levofloxacin	Isoniazid, Rifampin, Ethambutol, Clarithromycin, Levofloxacin	Yes	No	Alive
20	R	S	R	S	S	Isoniazid, Ethambutol, Amikacin, Levofloxacin	Isoniazid, Ethambutol, Amikacin, Levofloxacin	Yes	Abdominal surgery, Lymphadenectomy	Alive
21	N/D	N/D	N/D	N/D	N/D	Isoniazid, Rifampin, Ethambutol	Rifampin, Isoniazid, Ethambutol	No	No	Alive
22	S	S	S	S	S	Isoniazid, Rifampin, Ethambutol, Ciprofloxacin	Rifampin, Isoniazid, Ethambutol, Levofloxacin, Amikacin	No	No	Alive
23	S	S	S	N/D	N/D	Isoniazid, Rifampin, Ethambutol, Levofloxacin	Isoniazid, Rifampin	No	Lymphadenectomy	Alive
24	S	S	S	N/D	N/D	Rifampin, Isoniazid, Levofloxacin, Amikacin	Rifampin, Isoniazid, Levofloxacin, Amikacin	No	Abdominal surgery, Lymphadenectomy	Deceased (Severe BCG infection)

~ Used for a short time and discontinued

AMIK; amikacin, CIP; ciprofloxacin, ETB; ethambutol, N/D; not determined, INH; isoniazid, RIF; rifampin, S; sensible, R; resistant

### Treatments and outcomes

All patients received three or more anti-mycobacterial agents which were then refined according to the susceptibility profile or clinical response. All agents were used in their oral form, except for amikacin which was given intravenously and linezolid which was started intravenously and then orally. Table 2 summarizes the initial empiric treatment and eventual anti-mycobacterial regimens that were applied in patients.

All except three patients (P11, P18, and P21) were initiated on second-line anti-mycobacterial agents with either a fluoroquinolone (levofloxacin: 15 mg/kg/day, ciprofloxacin: 20 mg/kg/day, ofloxacin: 15 mg/kg/day), aminoglycoside (amikacin: 10–15 mg/kg/day, streptomycin: 15 mg/kg/day), and/or macrolide (clarithromycin: 15 mg/kg/day) along with oral rifampin (10 mg/kg/day), isoniazid (15 mg/kg/day), and ethambutol (20 mg/kg/day).

Four patients (16.7%) received a three-drug anti-mycobacterial regimen as empiric therapy with rifampin, ethambutol, and isoniazid/pyrazinamide. After identification of *M. bovis*-BCG, pyrazinamide was discontinued in three patients (12.5%). Three other patients (P1, P11, and P18) showed a clinical response to rifampin, despite in vitro resistance obtained in the susceptibility test. Other most frequent second-line anti-mycobacterial agents included cycloserine (15–20 mg/kg/day), prothionamide (15–20 mg/kg/day), and linezolid (10 mg/kg/day) which were used as part of the main regimen or for a short period of time in 7 (29.2%), 4 (16.7%), and 3 (12.5%) patients, respectively. 14 (58.3%) patients received adjuvant subcutaneous injections of IFN-γ, 50 µ/m^2^ every other day. 13 (54.2%) patients required surgical operations including lymphadenectomy, central vein catheterization, diagnostic laparotomy (prior to the diagnosis of MSMD), intestinal segment resection (due to the obstruction, and psoas abscess drainage in one patient (Table 1). At the end of the survey, 22 out of 24 patients were alive and two patients (P1 and P24) died due to BCG-osis and respiratory failure.

## Discussion

The selection of an appropriate antimycobacterial regimen for BCG disease, particularly in patients with IEI, MSMD in particular, is still a challenging issue as there is no evidence-based treatment guideline, and decisions are made on a case-by-case basis. In this regard, we retrospectively summarized our experience with antimycobacterial therapeutic protocols applied for MSMD patients with BCG-osis and related outcomes.

Once the diagnosis of BCG-osis is established, even before determining underlying genetic defect, a trial of at least three or four antimycobacterial antibiotics is recommended in local BCG infection with regional lymphadenopathy and disseminated forms, respectively [19, 20].

In 2016, the world health organization (WHO) changed its therapeutic policy for multi-drug resistant tuberculosis (MDR TB) and introduced fluoroquinolones, including levofloxacin, moxifloxacin, and gatifloxacin as the first line anti-mycobacterial agents [21]. In the present study, all BCG-osis patients showed clinical improvement with the addition of second-line anti-mycobacterial medications. Therefore, for BCG-osis we also suggest a similar approach in early instigation of second-line anti-mycobacterial agents, particularly fluoroquinolones and aminoglycosides (such as amikacin).

The number of MSMD patients who were reported to receive second-line anti-mycobacterial agents as initial treatment of BCGosis is limited. In 2017, Boudjemaa et al. presented two French siblings with delayed growth, generalized granulomatous osteomyelitis, and inflammatory state. In one sibling, regional fistulized post-vaccination adenitis was also reported. BCG-osis was confirmed by the detection of bacilli in the fine needle biopsy sample of bone lesions. Both patients were finally diagnosed with MSMD with STAT1 deficiency and received a combination of moxifloxacin, ethambutol, isoniazid, rifampicin, and IFN-γ, leading to dramatic clinical improvement [22]. Two unrelated Arab patients with BCG-osis also received fluoroquinolone as part of the antimycobacterial treatment. One patient with a history of post-vaccine adenitis, later complicated with generalized lymphadenopathies, pleural effusion, and ascites. The BCG-osis diagnosis was confirmed by finding numerous acid-fast bacilli in the lymph node biopsy and GW test and *M. bovis*-BCG strain in the culture. Based on the drug susceptibility test, he finally received ethambutol, amikacin, capreomycin, moxifloxacin, para-aminosalicylic acid, and linezolid together with IFN-γ and achieved stable clinical condition. Another patient with BCG vaccine complication, multiple episodes of lymph node enlargement and candidiasis, was found to have *M. bovis*-BCG resistant to isoniazid and rifampin. She finally received amikacin, rifampin, moxifloxacin, cycloserine, ethambutol, clarithromycin, and IFN-γ and gradually improved [23]. Due to the insufficient experimental evidence in the literature, individualized treatment decisions should be made on a case-by-case basis, as the cases are not comparable regarding the underlying etiology of BCG disease, severity of disease, and resistance pattern of the pathogens.

On the contrary, some studies reported patients with other IEIs different from MSMD who were prescribed fluoroquinolones together with first-line anti-mycobacterial agents but did not show significant improvement [24, 25]. An Iranian patient with a history of post-vaccine lymphadenitis, presented with thoracic abscess, abdominal ascites, splenomegaly, and abdominal lymphadenopathies. He was found to have positive gastric washing (GW) test for mycobacteria and abnormal LTT for BCG. Symptoms recurred after administration of anti-mycobacterial regimen, therefore, levofloxacin and IFN-γ were added. However, he later developed rifampin-resistant brain TB abscess and eventually diagnosed with IL-12Rβ1 deficiency [24]. In another study [25], 10 immunocompromised patients (5 MSMD, 4 SCID and one HIV patients) with disseminated BCG disease used moxifloxacin mostly with ethambutol and clarithromycin. Although most patients (80%) survived, this regimen did not alter the clinical condition in the majority (60%). As a conclusion, for the treatment of BCG-osis particularly in patients with immunodeficiency background, the timely selection of appropriate antibiotics can be life-saving and further studies are required to confirm our preliminary findings.

In communities with a high burden of tuberculosis, early diagnosis and treatment of patients with MSMD and other TB-related IEIs seem to be another challenge as most of the cases may be overlooked by the high rates of TB epidemics and patients may be in need of multiple courses of anti-TB drugs. In a recent multicenter study on 55 Indian patients with various forms of MSMD, almost 15% of patients required two or more courses of anti-TB medications, and patients with MDR TB required additional second-line drugs for a longer duration [26]. Furthermore, most of the endemic countries lack or have limited resources to apply next-generation sequencing in patients suspected to have defects in the IL-12-IFN-γ pathway. Recently, van Coller et al. have suggested the implementation of functional immunoassays in patients with suspected IEIs relating to mycobacterial susceptibility, particularly in TB endemic regions [27]. In addition, some of the patients with BCGosis are later turn out to be SCID and other IEIs susceptible to fungal infections. To build an effective anti-TB regimen in such patients, probable drug interactions with azole antifungals should be addressed and second-line anti-TB medications can be used interchangeably [28].

More than half of the study population (58.3%) received subcutaneous IFN-γ together with the anti-mycobacterial regimen. However, they did not receive it regularly and we could not assess its efficacy. Adjuvant IFN-γ therapy can restore macrophage function and provide better control of BCG-osis in patients with CGD and MSMD [29], which seems to particularly improve the prognosis for patients with IL-12Rβ1 deficiency [30]. However, it is ineffective in the absence of functional surface receptors observed in autosomal recessive complete IFN-γR1 and IFN-γR2 deficiency [14].

For patients with partial IFN-γR deficiency after the resolution of the acute infection, lifelong administration of azithromycin can prevent the recurrence of infections. Nonetheless, in patients with *IL12B* mutations with milder phenotype, the need for a prophylactic antibiotic is debated [31] and may be restored for those with recurrent *Salmonella* infection. Currently, there is no consensus on the utility of prophylactic antibiotics. However, it is suggested in patients with IL-12Rβ1 or IL-12p40 deficiency due to their high mortality and few other management options [32].

This study has several limitations, including retrospective design and the small number of clinically heterogeneous patients followed for almost a decade. Further prospective studies with a large study population and control group, among different categories of patients with inborn errors of immunity are required to develop comprehensive guidance for BCG disease which includes information on predispositions and recommended testing strategies.

Although we reported therapeutic protocols for the management of BCG-osis used in patients with MSMD, they can also be applied to other immunodeficiency backgrounds as well.

## Conclusion

The mainstay of treatment in BCG-osis is anti-mycobacterial agents and it should not be delayed until the genetic results of suspected underlying immunodeficiency become available. In patients with BCG-osis and underlying MSMD, the combination of antibiotics needs to be optimized (e.g., by microbiological studies of causative microorganisms at the early stage of the disease) to prevent upcoming side effects and toxicity, as most of them require prolonged administration of anti-TB medications probably even in periods of disease remission. In this regard, as with patients in this study, the early instigation of second-line anti-mycobacterial agents may be clinically beneficial and result in favorable outcomes.

## Supplementary Information

**Additional file 1.** Details of immunologic findings.

**Additional file 2.** Summaries of patients’ clinical history.

## Data Availability

All data generated or analyzed during this study are included in this published article [and its additional files].

## Abbreviations

AR: Autosomal recessive
BCG: Bacillus-Calmette Guérin
CGD: Chronic granulomatous disease
CRP: C-reactive protein
ESR: Erythrocyte sedimentation rate
HIV: Human immunodeficiency virus
HSCT: Hematopoietic stem cell transplantation
IEI: Inborn error of immunity
IQR: Interquartile range
LTT: Lymphocyte transformation test
MDR TB: Multi-drug resistant tuberculosis
MSMD: Mendelian susceptibility to mycobacterial disease
NBT: Nitro-blue tetrazolium
PPD: Purified protein derivative
rIFN-γ: Recombinant interferon-gamma
SCID: Severe combined immunodeficiency
TB: Tuberculosis
WES: Whole-exome sequencing
