# Supplementary material for: Effective anti-mycobacterial treatment for BCG disease in patients with Mendelian Susceptibility to Mycobacterial Disease (MSMD): a case series
Source: Ann Clin Microbiol Antimicrob. 2022 Mar 1;21:8. doi: 10.1186/s12941-022-00500-y (PMC8889629; doi:10.1186/s12941-022-00500-y)
Supplement: Supplementary file 1 — Additional file 1. Details of immunologic findings. [file 12941_2022_500_MOESM1_ESM.docx]

| **Table S1 – The summary of laboratory findings** | | | | | | | | | | | | | | | |
| --- | --- | --- | --- | --- | --- | --- | --- | --- | --- | --- | --- | --- | --- | --- | --- |
| **Pt No.** | **Age at evaluation** | **WBC ×10^3^ (cells/mm^3^)** | **Lymphocyte (%)** | **Neutrophil (%)** | **Eosinophil (%)** | **Monocyte (%)** | **CD3+ T cell (%)** | **CD4+ T cell (%)** | **CD8+ T cell (%)** | **CD19+ B cell (%)** | **CD16+ 56+ NK cell (%)** | **IgG (mg/dL)** | **IgA (mg/dL)** | **IgM (mg/dL)** | **IgE (mg/dL)** |
| 1 | 4.5 | 6.5 | 25% | 66% | 3% | 2% | 47% | 9% | 33% | 2% | 19% | 1470 | 165 | 100 | 160 |
| 2 | 5.3 | 15.5 | 33% | 60% | 1% | 6% | 48% | 32% | 18% | 3% | 16% | 1260 | 150 | 216 | 35 |
| 3 | 3.0 | 15.7 | 42% | 48% | 4% | 5% | 41% | 28% | 21% | 30% | 29% | 1750 | 175 | 89 | 9 |
| 4 | 4.0 | 7.5 | 34% | 51% | 5% | 10% | 36% | 29% | 12% | 28% | 19% | 482 | 90 | 98 | 400 |
| 5 | 0.9 | 16.6 | 48% | 25% | 1% | 5% | 42% | 18% | 27% | 16% | 20% | 1100 | 60 | 160 | 10 |
| 6 | 6.8 | 13.64 | 37% | 50% | 4% | 8% | 50% | 27% | 23% | 16% | 17% | 2105 | 288 | 61 | 89 |
| 7 | 6.9 | 5.4 | 56% | 67% | 2% | 6% | 62% | 39% | 19% | 22% | 6% | 1860 | 88 | 110 | 50 |
| 8 | 12.0 | 5.3 | 29% | 56% | 3% | 11% | 64% | 31% | 21% | 7% | 19% | 2700 | 159 | 360 | 100 |
| 9 | 2.5 | 11.4 | 64% | 26% | 5% | 4% | 42% | 19% | 19% | 25% | 12% | 694 | 22 | 460 | 45 |
| 10 | 9.5 | 10.0 | 42% | 53% | 2% | 3% | 35% | 12% | 28% | 38% | 6% | 1800 | 250 | 210 | 38 |
| 11 | 5.5 | 6.5 | 36% | 57% | 6% | 1% | 58% | 38% | 23% | 10% | 2% | 3850 | 570 | 400 | 51 |
| 12 | 0.2 | 16.7 | 62% | 21% | 6% | 8% | 63% | 26% | 44% | 25% | 12% | 1654 | 344 | 110 | 25 |
| 13 | 0.04 | 2.4 | 48% | 28% | 12% | 8% | 56% | 18% | 33% | 23% | 9% | 1600 | 321 | 340 | 1 |
| 14 | 3.0 | 3.3 | 31% | 56% | 3% | 8% | 60% | 12% | 35% | 20% | - | - | 140 | - | - |
| 15 | - | - | - | - | - | - | - | - | - | - | - | - | - | - | - |
| 16 | 11.0 | 18.0 | 11% | 86% | 3% | 2% | 60% | 33% | 19% | 11% | 10% | 2995 | 237 | 164 | 227 |
| 17 | 0.8 | 19.8 | 49% | 20% | 4% | 6% | 57% | 31% | 22% | 13% | 4% | 732 | 132 | 273 | 593 |
| 18 | 12.0 | 3.5 | 24% | 71% | 2% | 3% | 47% | 31% | 12% | 38% | 5% | 929 | 134 | 245 | 18 |
| 19 | 2.0 | 11.2 | 26% | 68% | 3% | 4% | 44% | 8% | 38% | 14% | 10% | 602 | 30 | 52 | 367 |
| 20 | 11.0 | 7.8 | 13% | 77% | 3% | 7% | 37% | 13% | 18% | 25% | 24% | 2979 | 157 | 154 | 64.5 |
| 21 | 4.0 | 11.3 | 20% | 63% | 1% | 8% | 52% | 30% | 18% | 20% | 25% | 2473 | 555 | 348 | 6 |
| 22 | 4.9 | 19.1 | 40% | 55% | 1% | 4% | 64% | 42% | 20% | 17% | 5% | 1920 | 220 | 150 | 36 |
| 23 | 0.8 | 12.6 | 79% | 16% | 3% | 2% | 69% | 40% | 23% | 14% | 6% | 780 | 69 | 59 | 5 |
| 24 | 1.3 | 7.3 | 68% | 27% | 2% | 8% | 48% | 2.5% | 30% | 9% | 10% | 2780 | 150 | 820 | 15 |
| *WBC; white blood cell, Ig; immunoglobulin* | | | | | | | | | | | | | | | |
