# Supplementary material for: Effective anti-mycobacterial treatment for BCG disease in patients with Mendelian Susceptibility to Mycobacterial Disease (MSMD): a case series
Source: Ann Clin Microbiol Antimicrob. 2022 Mar 1;21:8. doi: 10.1186/s12941-022-00500-y (PMC8889629; doi:10.1186/s12941-022-00500-y)
Supplement: Supplementary file 2 — Additional file 2. Summaries of patients’ clinical history. [file 12941_2022_500_MOESM2_ESM.docx]

**SUPPLEMENTARY ONLINE MATERIALS**

**CASE PRESENTATIONS:**

**Patient 1:**

P1 was a 5 years old Azeri male born to consanguineous parents. His older sister had BCG-adenitis after vaccination which had been resolved spontaneously. He developed axillary lymphadenopathy after BCG vaccination in his early infancy. He later developed hepatosplenomegaly and abdominal lymphadenopathy. The basic immunologic workup yielded normal results. The PPD skin test was negative. The gastric washing test was positive for mycobacteria species. He was diagnosed with BCG-osis and received isoniazid, rifampin, ethambutol, clarithromycin, ofloxacin, and IFN-ɣ, 50 µ/m^2^ every other day. He underwent lymphadenectomy and intestinal segment resection. About 18 months later clarithromycin was discontinued and dapsone and cycloserine were added to the regimen. The genetic study was performed and a homozygous missense mutation, c.344G>A (p.C115Y), in *IL23R* was identified by WES and confirmed by Sanger sequencing [16, 28].

Unfortunately, he did not respond to treatment and died at 8 years and 3 months due to BCG-osis and respiratory failure.

**Patient 2:**

P2 was a 10 years old Baluch female born to consanguineous parents. She was admitted to the hospital as she developed lymphadenitis after BCG vaccination at 7 months. She also suffered from oral candidiasis. The basic immunologic workup was normal. She was treated with an antimycobacterial drug regimen, including isoniazid, rifampin, ethambutol, and pyrazinamide for six months. She did not use her medication for one year and re-admitted to the hospital with the dissemination of infection. Treatment with isoniazid, rifampin, ethambutol, amikacin, clarithromycin, and IFN-ɣ (50 µ/m^2^ every other day) was restarted and two years later clarithromycin was replaced with ofloxacin. The WES revealed a homozygous Indel-frameshift mutation, c.527_528delCT (p. S176Cfs*12) at exon 5 of the *IL12B* gene, which was verified by Sanger sequencing. Both parents were heterozygous for this mutation [16].

**Patient 3:**

P3 was an 11.5 years old Persian female born to consanguineous parents. She received the BCG vaccine at birth and two months later developed bilateral axillary lymphadenopathy. At the age of 6 months, she was admitted to the hospital for supraclavicular lymphadenopathy, and treatment with isoniazid, rifampin, ethambutol, clarithromycin, and ofloxacin initiated. She also received a short period of amikacin and ethionamide (from the age of 6 months until 1.5 years). At the age of 1.5, she presented with fever, anorexia, cough, myalgia, hypertension, and weight loss. She was complicated with pleural effusion, and mediastinal and abdominal lymphadenopathy. PCR result for the MTB complex gastric washing and BCG in urine, pleural fluid, and gastric washing was positive. Isoniazid and rifampin discontinued, ofloxacin was replaced by levofloxacin, and treatment with clarithromycin and ethambutol continued. Amikacin was initiated again one year later. At 3.5 years, amikacin was discontinued and IFN-ɣ (50 µ/m^2^ every other day), cycloserine, prothionamide, and ethionamide were added to the regimen. At 4.5 years, AFB was isolated following bone marrow aspiration, prothionamide discontinued and treatment with linezolid and clofazimine started. She also presented salmonellosis and urinary *Klebsiella* infections. A homozygous missense mutation in *IL12R*$B1$, c.512A>C (p. Q171P), was identified through PCR amplification and sequencing. Both parents were heterozygous for this mutation [16, 22].

**Patient 4:**

P4 was a 7 years old Baluch female born to consanguineous parents. She was vaccinated with BCG at birth and about two weeks later developed peripheral lymphadenopathy. She had normal immunologic parameters as well as negative PPD and gastric washing tests. She also had oral candidiasis. She underwent abdominal lymphadenectomy. Treatment with antimycobacterial agents including isoniazid, rifampin, ethambutol, clarithromycin, ciprofloxacin, and IFN-ɣ (50 µ/m^2^ every other day) started. After one year she presented with salmonellosis. Ciprofloxacin was discontinued and levofloxacin and amikacin were added to the regimen. A genetic study revealed a homozygous Indel-Frameshift mutation at exon 5 of the *IL12B* gene, c.527_528delCT (p. S176Cfs*12), confirmed by Sanger sequencing [16].

**Patient 5:**

P5 was a 11 years old Persian male born to consanguineous parents. He received BCG vaccination at birth and presented with peripheral lymphadenopathy one month later. The PPD skin test was negative, but the gastric washing test was positive for BCG. The immunologic workup was normal. He was diagnosed with BCG-osis and treated with isoniazid, rifampin, ethambutol, clarithromycin, and ofloxacin for one year. The genetic evaluation identified a homozygous missense mutation at exon 7 of the *IL12R*$B1$ gene, c.635G>A (p. R212Q) [16].

**Patient 6:**

P6 was a 5-year-old Baluch girl born to non-consanguineous parents. She developed bilateral axillary lymphadenitis at 5 months old and was diagnosed with BCG-osis. The skin biopsy culture and gastric washing test were positive. The immunologic workup yielded normal results. She received ethambutol, rifampin, clarithromycin and pyrazinamide and later isoniazid, levofloxacin, and IFN-ɣ (50 µ/m^2^ every other day) were added to the regimen. She was finally found to have a homozygous Indel-Frameshift mutation at exon 5 of the *IL12B* gene, c527_528delCT (p. S176Cfs*12) [16].

**Patient 7:**

P7 was a 10-year-old Persian male born to non-consanguineous parents. Four months after vaccination with BCG, he was referred to our hospital due to axillary lymphadenitis and three months later underwent lymphadenectomy. At the age of 2, he presented with pyrexia, weight loss, and sweating. The gastric washing culture was positive for BCG. He was treated with ethambutol, levofloxacin, prothionamide, and cycloserine for one year (and amikacin for 6 months) and continued with isoniazid, ethambutol, and levofloxacin for the second year. He is now well with no other infectious complications or relapse. The genetic study revealed a homozygous splice site mutation, c.367+1G>A, at the exon 3 of the *IL23R* gene [16].

**Patient 8:**

P8 was a 20.5 years old girl born to consanguineous parents. Following vaccination with BCG at birth, she developed abdominal and peripheral lymphadenopathy. She also presented disseminated rash which was found to be leukocytoclastic vasculitis. The anti-mycobacterial regimen started with isoniazid, rifampin, ethambutol, amikacin, clarithromycin, ofloxacin, and IFN-ɣ (50 µ/m^2^ every other day). She underwent abdominal surgery and lymphadenectomy. Dapsone and clofazimine were also prescribed for 6 months (2-2.5 years). Rifampin and isoniazid after 1 year and ofloxacin after 3.5 years were discontinued and replaced by prothionamide and cycloserine (at the age of 1.5 years), and levofloxacin and fluvoxamine (at the age of 3.5 years). The genetic study identified a homozygous Indel-frameshift mutation, c.527_528delCT (p. S176Cfs*12), at the exon 5 of the *IL12B* gene [16].

**Patient 9:**

P9 was a 9-year-old girl born to consanguineous parents. After BCG vaccination she presented with left axillary and later left subclavicular mass. The lymph node biopsy was positive for mycobacterium species and she underwent lymphadenectomy. She was complicated with hepatosplenomegaly and seizure. She received isoniazid, rifampin, ethambutol, clarithromycin, ciprofloxacin, and IFN-ɣ (50 µ/m^2^ every other day). The genetic evaluation revealed a homozygous essential splicing site mutation, c.783+1G>A, in the *IL12RB1* gene [16].

**Patient 10:**

P10 is a now 15-year-old Baluch male born to consanguineous parents. He presented with cervical and axillary lymphadenopathy was following BCG vaccination at early infancy. He was treated with isoniazid (for 3 years), rifampin (for 3 years), ethambutol (for 3 years), ofloxacin (for 2 years), amikacin (for 2 years), and clarithromycin (for 3 years). Streptomycin and prothionamide were also administered for 1 year. The BCG infection extended to the lung, brain, bone, joints, and he developed paravertebral and psoas abscesses with fistulization. Abscess drainage was performed and levofloxacin (for 4 years), amikacin (for 3.5 years), cycloserine (for 3 years), ethionamide (for 4 years), clofazimine (for 3 years), meropenem, co-amoxiclav (for 3 years), and IFN-ɣ (50 µ/m^2^ every other day) were added to the regimen. The genetic evaluation showed a homozygous nonsense mutation at exon 5 of the *TYK2* gene, c.462G>T (p.E154*) [16, 29].

**Patient 11:**

P11 is an 11 years old male born to consanguineous Persian parents. His family history was positive for the early death of an unknown cause in one of his cousins. He initially presented at 3 months of age with multiple lymphadenopathies at the axillary and subclavicular region. He also suffered from polyarticular swelling in the upper and lower extremities and also clubbing. The immunologic profile was normal, but gastric washing test and lymph node biopsy were positive for BCG. He was started on isoniazid, rifampin, ethambutol, and pyrazinamide but did not receive the medications regularly (He stopped them from 6 months to 3 years and from 3.5 years to 5 years). His manifestations recurred and finally, he was placed on isoniazid, rifampin, ethambutol, co-trimoxazole, and IFN-ɣ (50 µ/m^2^ every other day) for 3 years. Amikacin was also started but after one year, he showed ototoxicity to amikacin; therefore, it was replaced by levofloxacin and continued for 2 years. In the genetic study, previously described essential splicing site mutation was found in the *IL12RB1* gene (c.1791+2T˃G) [16].

**Patient 12:**

P12 is an 8-year-old male born to non-consanguineous parents. He presented with axillary, cervical, and inguinal lymphadenopathy and abdominal distension. Imaging studies showed mediastinal and para-aortic lymphadenopathy and also hepatomegaly. BCG-osis was diagnosed and an anti-mycobacterial regimen including isoniazid, rifampin, ethambutol, and clarithromycin was started and continued for six years. The patient also received IFN-ɣ (50 µ/m^2^ every other day) and a six-month period of cycloserine (6 months to 1 year old). The genetic study was performed and a nonsense mutation at *IL12RB1* gene, c.847C>T (p.R283*), confirmed the diagnosis of MSMD [16].

**Patient 13:**

P13 is a 9.5 years old female born to close-relative parents. She presented at 4 months of age with complaints of fever and weight loss. In the physical examination, axillary and cervical lymphadenopathy, hepatosplenomegaly, and erythema at the site of BCG vaccine injection were observed. In the lymph node biopsy, necrotizing granulomatous inflammation with numerous acid-fast bacilli was identified. The diagnosis of BCG-osis was established. She underwent lymphadenectomy and received isoniazid, rifampin, ethambutol, ofloxacin, and clarithromycin. After one year, ethambutol, ofloxacin, and clarithromycin were discontinued but other medications were used for 8 years. The genetic study showed an essential splicing site mutation (c.783+1G>A) at intron 8 of the *IL12RB1* gene [16].

**Patient 14:**

P14 is a 16.5-year-old male, referred to our hospital at 7 years old with recurrent pneumonia, otitis, oral candidiasis, and also failure to thrive. His parents were relatives and he had a history of generalized lymphadenopathy and hepatosplenomegaly at 3 months of age. In the abdominal CT scan, intestinal loop thickening and paraaortic lymphadenopathy were observed. The immunologic profile was normal. The gastric washing test was positive. The treatment with antimycobacterial agents was initiated including isoniazid, rifampin, ethambutol, clarithromycin, and ofloxacin and continued for seven years. Meanwhile, the genetic study was performed and resulted in the finding of an Indel-frameshift mutation at the *IL12B* gene, c.527_528delCT (p. S176Cfs*12) [16].

**Patient 15:**

P15 is a 10-year-old male born to consanguineous parents. He presented at 7 months old with right axillary lymphadenopathy started at 2 months of age, abdominal distension, and failure to thrive. In ultrasound, multiple abdominal lymphadenopathies and splenomegaly were detected. He underwent lymphadenectomy and biopsy was positive for acid-fast bacilli. He received an antimycobacterial regimen including rifampin, isoniazid, ethambutol, ofloxacin, and IFN-ɣ (50 µ/m^2^ every other day). One year later, he presented with left-side scrotal swelling and erythema, surgically investigated and found to be lymphadenopathy. In the genetic investigation, a novel Indel-frameshift mutation (c. 850_851insACAG, p. L284Hfs*12) at the *IL12RB1* gene was found [16].

**Patient 16:**

P16 was a 12-year-old male who was born to consanguineous parents. He was the second child of the family and his siblings are healthy. When he was six months old, he presented with fever and axillary lymphadenopathy and received isoniazid for six months. About seven months ago, he developed bilateral cervical lymphadenopathy with injection. The histopathologic examination revealed granulomatous reaction and a few large CD30 positive lymphoid cells, suspicious of lymphoma. The immunologic workup yielded normal findings. He was complicated with a neck abscess and required drainage. In the whole-exome sequencing, a homozygous mutation in the *IL12B* gene was identified, conferring a complete IL-12p40 deficiency. Treatment with isoniazid (for 1.5 years), rifampin (for 1.5 years), ethambutol (for 1.5 years), and clarithromycin (for 3 months) was started and after 3 months, levofloxacin and amikacin were added to the regimen and continued for 1.5 years.

**Patient 17:**

P17 is a 3-year-old Afghan male born to nonconsanguineous parents. At 27 months old, he presented with complaints of persistent axillary lymphadenopathy in the past two years, recurrent respiratory tract infections, recurrent otitis, and cutaneous maculopapular lesions predominantly on the face and cervical region. He also suffered from hepatosplenomegaly, seizure, and mild pulmonary hypertension. In the abdominal ultrasound, paraaortic lymphadenopathies with central necrosis were found. He was further complicated with *Klebsiella* and *Enterobacter* infections. Treatment with isoniazid, rifampin, ethambutol, levofloxacin, amikacin was started and after 6 months, cycloserine and IFN-ɣ (50 µ/m^2^ every other day) was added and all were continued for 1.5 years.

**Patient 18:**

P18 is a 13.5-year-old boy born to consanguineous parents. The family history is unremarkable except for the BCG-osis in his cousin. He had a history of BCG lymphadenitis at six months old and received isoniazid for six months. He was hospitalized at the age of one-year-old due to abdominal distension and fistulized lymphadenopathy and underwent lymphadenectomy. At the age of four years old, he presented again with abdominal distension and cough. He was complicated with bilateral hilar lymphadenopathy and hepatosplenomegaly. No oral candidiasis was detected. The immunoglobulin levels, lymphocyte subsets, and nitro blue tetrazolium (NBT) test were normal. He received an anti-mycobacterial regimen with rifampin, isoniazid, and ethambutol. At 8 years old, the gastric washing test yielded a positive result and he was found to be resistant to rifampin. In the last admission, he presented with fever, weight loss, sub-mandibular, and neck lymphadenopathy with infectious discharge. The neck mass excisional biopsy findings showed inflammatory granulation tissue contains numerous macrophage and neutrophils compatible with abscess formation.

**Patient 19:**

P19 is a 3.5 years old female who presented with left supraclavicular and axillary lymphadenopathy two months after birth. She received isoniazid and rifampin until 6 months of age. Two months later, she developed severe diarrhea, abdominal distension, and peripheral edema. The gastric washing test was positive and anti-TB regimen with isoniazid, rifampin, ethambutol, and IFN-ɣ (50 µ/m^2^ every other day). She was referred to our center due to protracted diarrhea and hypoalbuminemia and was diagnosed with protein-losing enteropathy. The regimen was changed to rifampin, isoniazid, ethambutol, levofloxacin, and clarithromycin, and monthly infusion of albumin.

**Patient 20:**

P20 is a 12-year-old female first child of Azeri consanguineous parents. She primarily manifested with left axillary and supraclavicular lymphadenopathies at two months of age. At 4 years old, she received an antimycobacterial regimen with isoniazid, ethambutol, amikacin, levofloxacin, and IFN-ɣ (50 µ/m^2^ every other day) for 1 year. She also had symptoms of allergic rhinitis and was complicated with septic arthritis and pneumonia. After 6 years, she presented fistulae with infectious discharge on the abdomen. In ultrasound, abdominal lymphadenopathies and splenomegaly were observed. She underwent abdominal surgery and lymphadenectomy. The genetic evaluation revealed a homozygous mutation in the *IL12RB1* gene (c.G1193C, p.W398S) and she was diagnosed with MSMD.

**Patient 21:**

P21 is a 5 years old female, sibling of P20. Due to the positive family history, she did not receive the BCG vaccine. At 3 years old, she was hospitalized for persistent cough and bloody diarrhea and her gastric washing test reported a positive result for *M. bovis*-BCG. She also had pericardial effusion and a pericardial window was inserted. At 4 years old, she presented with left submandibular lymphadenopathy and was finally diagnosed with MSMD and as like her sister a homozygous mutation in the *IL12RB1* gene (c.G1193C, p.W398S) was identified. She received rifampin, isoniazid, and ethambutol for 1 year.

**Patient 22:**

P22 was an 8-year-old female born to consanguineous parents. Her older brother had been complicated with axillary lymphadenopathy, oral candidiasis, and severe protracted diarrhea after BCG vaccination and died at five years old. Therefore, she did not receive the BCG vaccine. At 2.5 years old, he developed bilateral submandibular and right inguinal lymphadenitis. The PPD skin test was negative but the gastric washing test was positive. She was diagnosed with BCG-osis and the anti-mycobacterial regimen was initiated with rifampin, isoniazid, and ethambutol, and later amikacin and levofloxacin were added on. She was found to have a homozygous mutation in *IL12RB1* gene, p.R173W,

**Patient 23:**

P23 is a 4-year-old male with distant-relative parents. Four months after BCG vaccination, he presented with left axillary and cervical lymphadenopathy and fistulized abscess on the left arm. He was diagnosed with localized BCGitis. He underwent lymphadenectomy and received isoniazid, rifampin, ethambutol, and levofloxacin.

**Patient 24:**

P24 was a one and half-year-old female born to close relative parents. Her siblings were healthy. At the age of three months old, she presented with axillary lymphadenopathy and oral candidiasis. In the gastric washing test, mycobacterium BCG was detected and she was diagnosed with BCG-osis. She was referred to our hospital with complaints of cough and wheezing. At the chest CT scan, mass-like pulmonary consolidation and necrotizing lymphadenitis were found. After two weeks of anti-BCG treatment with isoniazid, rifampin, levofloxacin, and amikacin in our center, unfortunately, she passed away due to severe BCG-osis. A WES was performed but no mutation was found in known genes involved in MSMD.
